# L-Arginine supplementation inhibits the growth of breast cancer by enhancing innate and adaptive immune responses mediated by suppression of MDSCs in vivo

**DOI:** 10.1186/s12885-016-2376-0

**Published:** 2016-06-01

**Authors:** Yu Cao, Yonghui Feng, Yanjun Zhang, Xiaotong Zhu, Feng Jin

**Affiliations:** Department of Surgical Oncology and Breast Surgery, The First Affiliated Hospital of China Medical University, Shenyang, Liaoning 110001 China; Department of Laboratory Medicine, The First Affiliated Hospital of China Medical University, Shenyang, Liaoning 110001 China; Department of Medical Examination Center, The First Affiliated Hospital of China Medical University, Shenyang, Liaoning 110001 China; Department of Immunology, College of Basic Medical Sciences, China Medical University, Shenyang, Liaoning 110122 China

**Keywords:** L-Arginine, Breast cancer, Tumor immunity, MDSCs

## Abstract

**Background:**

L-Arg is involved in many biological activities, including the activation of T cells. In breast cancer patients, L-Arg is depleted by nitric oxide synthase 2 (NOS2) and arginase 1 (ARG-1) produced by myeloid-derived suppressor cells (MDSCs). Our aim was to test whether L-Arg supplementation could enhance antitumor immune response and improve survivorship in a rodent model of mammary tumor.

**Methods:**

Tumor volumes in control and L-Arg treated 4 T1 tumor bearing (TB) BALB/c mice were measured and survival rates were recorded. The percentages of MDSCs, dendritic cells (DCs), regulatory T cells (Tregs), macrophages, CD4^+^ T cells, and CD8^+^ T cells were examined by flow cytometry. Additionally, levels of IL-10, TNF-α, and IFN-γ were measured by enzyme-linked immunosorbent assay (ELISA) and nitric oxide (NO) levels were measured by the Griess reaction. IFN-γ, T-bet, Granzyme B, ARG-1 and iNOS mRNA levels were examined by real-time RT-PCR.

**Results:**

L-Arg treatment inhibited tumor growth and prolonged the survival time of 4 T1 TB mice. The frequency of MDSCs was significantly suppressed in L-Arg treated TB mice. In contrast, the numbers and function of macrophages, CD4^+^ T cells, and CD8^+^ T cells were significantly enhanced. The IFN-γ, TNF-α, NO levels in splenocytes supernatant, as well as *iNOS*, IFN-γ, Granzyme B mRNA levels in splenocytes and tumor blocks were significantly increased. The ARG-1 mRNA level in tumor blocks, the frequency of Tregs, and IL-10 level were not affected.

**Conclusion:**

L-Arg supplementation significantly inhibited tumor growth and prolonged the survival time of 4 T1 TB mice, which was associated with the reduction of MDSCs, and enhanced innate and adaptive immune responses.

## Background

Despite advances in multimodal therapies, breast cancer remains a significant problem that causes deaths in women worldwide. Although the incidence and mortality vary by geographical region, the overall incidence of breast cancer is increasing [1]. Breast cancer is often associated with immune suppression in humans and L-arginine (L-Arg) depletion, an occurrence which can be effectively modeled in tumor-bearing (TB) mice. Therefore, it is necessary to identify new therapeutic targets for the breast cancer, especially for the regulation of immune responses.

L-Arg is an essential amino acid for infants and young children but a conditionally essential amino acid for adults. It can be metabolized into nitric oxide (NO) and L-citrulline by inducible nitric oxide synthase (iNOS) or into urea and L-ornithine by ARG-1 [2]. NO modulates different cancer-related events. However, several lines of research have indicated that NO may have dual effects in cancer [3]. L-Arg plays a central role in several biologic systems, including the activation of T cell function [4]. L-Arg depletion by myeloid-derived suppressor cells (MDSCs), which produce arginase 1 (ARG-1) and NO synthase 2 (NOS2), is observed in cancer patients [2, 5–7]. This subset of myelomonocytic cells promotes tumor growth and metastasis, which are highly efficient at suppressing activated T cells, leading to the impairment of general and tumor-specific adaptive immune responses [2, 8]. Activated T cells cultured in a medium without L-Arg, or cocultured with ARG-1 producing MDSCs isolated from tumors, proliferate at a decreased rate, express lower levels of the T cell receptor CD3 chain, and produce reduced levels of cytokines [6, 9, 10]. Such impaired T cell functions can be reversed by the enteral or parenteral supplementation of L-Arg [11].

Considering that (1) L-Arg level is decreased in tumor bearing patients and mice [12], (2) anti-tumor T cell immunity is usually suppressed, whereas MDSCs which mediate tumor escape are always enhanced in the tumor bearers, and (3) L-Arg depletion by MDSCs leads to the depression of T cells [7, 13], we hypothesized that L-Arg supplementation would inhibit tumor growth and improve survival. Murine models have been established to study breast cancer focusing on the specific clinical questions. In order to test this hypothesis, we supplemented the breast cancer-bearing BALB/c mice with L-Arg and monitored the host’s anti-tumor immune responses. Results showed that supplementation with L-Arg prolonged survival time of the host and inhibited tumor growth. This effect is associated with enhanced innate and adaptive immune responses. The results suggest that L-Arg supplementation may be a viable preventative and/or adjunctive treatment for the inhibition of breast cancer development.

## Methods

### Cell line and tumor implantation

The 4 T1 mouse mammary carcinoma cell line lacks the expression of estrogen receptor (ER) and metastasizes to other organs in a way similar to what is observed in naturally occurring breast cancer in humans [14], thus we selected 4 T1 mouse mammary carcinoma cell line to establish the breast cancer model. 4 T1 cell line was obtained from the Cell Bank of the Chinese Academy of Sciences (Shanghai, China). All animal experimental protocols were approved by the Animal Care and Use Committee of China Medical University. Cells were cultured in RPMI 1640 medium supplemented with 10 % fetal bovine serum (FBS) and 1 % penicillin-streptomycin, at 37 °C and 5 % CO_2_ in a 95 % humidified incubator. L-Arg was purchased from Sigma-Aldrich (St. Louis, MO, USA) and diluted to 150 mg/ml with phosphate-buffered saline (PBS), pH 7.0. Female, 6–8-week-old BABL/c mice were purchased from Academia Sinica Shanghai Experimental Animal Center (Shanghai, China). Mice were housed under controlled light and temperature conditions and randomly assigned to experimental and control groups of ten mice each. 4 T1 mouse mammary carcinoma cells (1 × 10^5^) were injected subcutaneously into the shaved flanks of mice. L-Arg treatment was then initiated on day 7 post-inoculation when the diameter of the tumor was palpable. For the dose selection of L-Arg used in the current study, we examined three different doses L-Arg (2.5 g/kg, 1.5 g/kg and 0.5 g/kg) supplementation on the tumor volume based on published literature [15, 16], and then 1.5 g/kg L-Arg was chosen to perform the following studies. Mice in the experimental group were treated for 20 consecutive days via oral administration of L-Arg (1.5 g/kg), whereas the control group received equal amounts of PBS once a day. To explore the role of NO, some mice were supplied with water with 1 % aminoguanidine (AG), an NOS inhibitor. Tumor growth was monitored every three days by measuring the tumor length (L) and width (W) using calipers and calculating the tumor size according to the following formula: Tumor volume (mm^3^) =1⁄2 × long diameter × short diameter squared. At the end of the treatment (day 28 post inoculation), three animals of each group were euthanized with ether. Tumor mass, lymph nodes, and spleens were removed for further analysis.

### Flow cytometry

Spleens and lymph nodes from 4 T1 TB BALB/c mice were dissected and homogenized to produce a single cell suspension. After red blood cells were lysed, the cells were washed with PBS (300 × g for 7 min) and adjusted to 1 × 10^7^/ml with RPMI-1640. Dendritic cells (DCs) were stained with FITC-anti-CD11c (clone HL3, BD Biosciences), PE-anti-CD11b (clone M1/70, BD Biosciences), PerCP-anti- CD45R⁄ B220 (clone RA3-6B2, BD Biosciences) and APC-MHC II (clone M5/114.15.2, eBioscience). To assess regulatory T cells (Tregs), FITC-anti-CD4 (clone H129.19, BD Biosciences) and PE-anti-CD25 antibodies (clone PC61, BD Biosciences) were added to spleen cells, and resuspended in 100 μl of PBS supplemented with 3 % FCS for surface staining. Then, the cells were fixed and permeabilized, and intracytoplasmic staining was performed using APC-anti-Foxp3 (clone FJK16s, eBioscience) antibody. For assessing CD4^+^ and CD8^+^ T cells, single cell suspensions from spleens and lymph nodes were stained with FITC-anti-CD3e (clone 145-2C11, BD Biosciences), PE-anti-CD4 (clone H129.19, BD Biosciences) and PerCp-anti-CD8α (clone 53–6.7, BD Biosciences). For the staining of macrophages and MDSCs, PerCP cy5.5-anti-F4/80 (clone BM8, ebiosciences), FITC-anti-CD11b (clone M1/70, BD Biosciences), and APC-anti-Gr-1 (clone RB6-8C5, BD Biosciences) were added into the 100 μl single splenocyte suspension and incubated for 30 min at 4 °C.

Intracellular cytokine staining assays were performed as described elsewhere [17]. Briefly, cells isolated from spleens were stimulated with PMA (50 ng/ml), ionomycin (1 μg/ml), and brefeldin (Sigma) in order to induce IFN-γ production. LPS (1 μg/ml) and GolgiPlugô were used to induce IL-12 production. Following stimulation, the cells were incubated for 5 h in RPMI 1640 medium containing 10 % FBS at 37 °C. Cells were collected and washed twice followed by surface staining as described above. The cells were then fixed and permeabilized with Cytofix/Cytoperm (BD Biosciences) according to the manufacturer's instructions and stained intracellularly with PE-anti-IFN-γ (clone XMG 1.2, BD Biosciences) and PE-anti-IL-12p40/70 (clone C15.6, BD Biosciences) or with corresponding isotope control antibodies for 30 min in permeabilization buffer (BD Biosciences). After washing twice in permeabilization buffer, the cells were resuspended in PBS.

For tumor staining and ROS measurement, tumors were removed from each mouse at the end of treatment and minced into small pieces and digested with 500 U/ml collagenase (type IV, Sigma) for 1 h at 37 °C with agitation. The resultant cells were passed through nylon mesh to remove debris, and viable cells were washed with PBS with 2 % FBS. Intracellular ROS generation was assessed using 2’,7,-dichlorofluorescein diacetate (DCFH-DA , Sigma). Briefly, 1 × 10^6^ cells were plated on the 6-well plates and incubated with DCFH-DA (10 mmol/L) for 30 min at 37°^C o^C and stained with corresponding antibodies as described above. After washing twice with PBS, the cells were resuspended in PBS containing 5 % FCS.

Data were acquired using FACS Calibur (BD Biosciences, San Diego, CA, USA) and analyzed using FlowJo v7.6.2 (Tree Star Inc., Ashland, OR, USA).

### Cytokine assays by ELISA and NO assay by Griess reaction

Splenocytes harvested from each group of mice were cultured for 48 h followed by collection of the supernatants. Levels of IFN-γ, TNF-α and IL-10 were determined with a corresponding ELISA kit (R&D Systems, Minneapolis, MN) according to the manufacturer’s instructions. To determine NO production, concentrations of NO_2_^−^ in cell supernatants were measured by the Griess reaction [18]. Briefly, 100 μl of the supernatant was incubated with 100 μl of Griess reagent [equal volumes of 1 % (w/v) sulfanilamide (Wako, Osaka, Japan) and 0.1 % (w/v) N-1-naphtyl ethylenediamine dihydrochloride (Wako) in 2.5 % (w/v) H_3_PO_4_] for 10 min at room temperature, and NO_2_^−^ concentration was determined by measuring the optical density at 550 nm (A550) in reference to the A550 of standard NaNO_2_ solution.

### RNA isolation and real-time RT-PCR

Total RNA of spleen cells (5 × 10^6^) and Tumor tissue (nearly 100 mg) was extracted by using Trizol (Invitrogen, Carlsbad, CA) according to the manufacturer’s instructions and quantified by OD at 260 nm using a UV-VIS spectrophotometer (PYE-UNICAM, USA). To remove genomic DNA contamination, RNA was treated with DNaseI and reverse-transcribed using a prime-script reagent kit (Takara Biotechnology, China). cDNA was synthesized using PrimeScript^TM^ RT reagent kit with gDNA Eraser (Takara). Reverse transcription was performed in a 10 μl reaction mixture containing PrimeScript^TM^ buffer, PrimeScript^TM^ RT enzyme mix, oligo dT primer (50 μM), random 6 mers (100 μM) and 500 ng of total RNA. PCR was performed with the resulting cDNA as a template and specific oligonucleotide primers. Primers used for the sequence-specific PCR were shown in Table 1. Quantitative PCR was carried out using a SYBR® Premix Ex Taq™ reagent kit (Takara) on ABI 7500 (ABI, USA). After denaturation at 95 °C for 30 s, 40 cycles of PCR (95 °C for 5 s and 60 °C for 30 s) were performed. Amplification of the *β-actin* sequence served as an internal control. Each experiment was performed three times independently. The average cycle threshold (CT) of the duplicate measurements was calculated. After verifying that amplification efficiencies of the target genes and *β-actin* were approximately equal, the 2^-ΔΔ^CT method was used to quantify the relative gene expression in the PBS group and experiment groups.

**Table 1:** Primer sequences for RT-PCR

Primer Name	Sequence (5’–3’)
β-actin_F	GATTACTGCTCTGGCTCCTAGC
β-actin_R	GACTCATCGTACTCCTGCTTGC
IFN-γ_F	GTTACTGCCACGGCACAGTCATTG
IFN-γ_R	ACCATCCTTTTGCCAGTTCCTCCAG
Granzyme B_F	CCTGAAGGAGGCTGTGAAAGAATC
Granzyme B_R	CCCTGCACAAATCATGTTTAGTCC
T-bet_F	TCAACCAGCACCAGACAGAG
T-bet_R	AAACATCCTGTAATGGCTTGTG
iNOS_F	TCCTCACTGGGACAGCACAGAATG
iNOS_R	GTGTCATGCAAAATCTCTCCACTGCC
ARG-1_F	ATGGAAGAGACCTTCAGCTAC
ARG-1_R	GCTGTCTTCCCAAGAGTTGGG

### Statistics analysis

Survival analysis was tested by the Kaplan–Meyer method. Results were expressed as the mean value ± SD and interpreted by Student’s *t-test*. Differences were considered statistically significant when *P <* 0.05.

## Results

### L-Arg supplementation slows the growth of 4 T1 breast carcinoma cells and prolongs survival

**Fig. 1:**
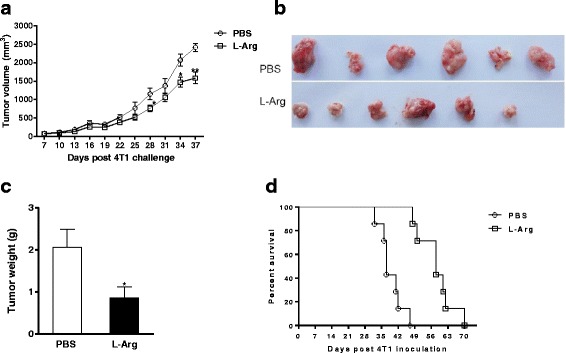
L-Arg treatment improved the survival of mice and inhibited the growth of 4 T1 mammary carcinoma in mice. (**a**) Growth curve of 4 T1 tumors between L-Arg and PBS groups. Tumor blocks (**b**) and weights (**c**) removed from the two groups at the end of treatment were compared. The survival (**d**) of 4 T1 TB mice (*n* = 7) was checked every day. * *P <* 0.05 and ** *P <* 0.01 compared to PBS group

To determine whether L-Arg supplementation in 4 T1 TB mice could improve the survival of mice upon tumor development, 4 T1 TB mice were orally administered with L-Arg for 20 consecutive days beginning when the tumor was palpable. We found that L-Arg supplementation could inhibit tumor growth in 4 T1 TB mice, and tumor volume in the L-Arg supplementation group was significantly smaller than the control group from day 28 on (*P <* 0.05, *t-test*) (Fig. 1a). Similarly, the tumor size and weight from the L-Arg treatment group was significantly reduced (*P <* 0.05, *t-test*) compared with that of the control group (Fig. 1b and c). In addition, there was a significant difference in the survival curve between L-Arg treatment and control groups (*P <* 0.01, Kaplan-Meier analysis). All mice in the PBS group died between day 32 and 47 post 4 T1 cell inoculation. In contrast, L-Arg treatment significantly prolonged the survival of 4 T1 inoculated mice, with death beginning at 48 days post 4 T1 inoculation (Fig. 1d).

### L-Arg suppresses the MDSCs from spleen and tumor

**Fig. 2:**
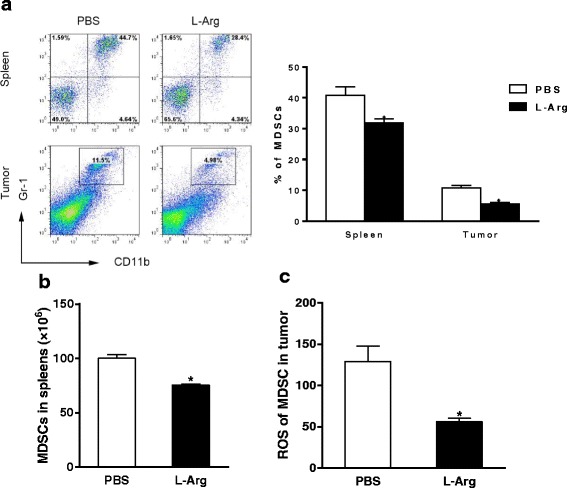
L-Arg suppressed MDSCs from spleens and tumors of 4 T1 TB mice. The frequencies and absolute numbers of MDSCs in the spleens and tumors (**a** and **b**) were assayed by FACS. Mean fluorescence intensity (MFI) (**c**) of ROS in MDSC obtained from tumor in 4 T1 TB mice. * *P <* 0.05 compared to PBS group

In TB mice, a heterogeneous mixture of myeloid cells expands at various stages of development. These cells may not only inhibit anti-tumor immunity but also directly stimulate tumorigenesis as well as tumor growth and expansion. This population efficiently suppresses T cell immune functions and is characterized primarily by expression of CD11b and Gr-1 [4]. Here, we compared the frequencies of MDSCs (CD11b^+^ Gr-1^+^) from the spleens and tumors in both L-Arg and control groups. As shown in Fig. 2a and b, oral administration of L-Arg significantly reduced the percentage of MDSCs from both splenic cells (*P <* 0.05, *t-test*) and tumor cells (*P <* 0.05, *t-test*) as well as the number of MDSCs in the spleen in 4 T1 TB mice. Meanwhile, L-Arg treatment significantly decreased the ROS levels in MDSCs in the tumor tissues (*P <* 0.05, *t-test*) (Fig. 2c). These results indicated that L-Arg supplementation may lead to enhanced anti-tumor immune responses.

### L-Arg supplementation promotes Gr-1^+^CD11b^−^F4/80^+^ macrophages but suppresses Gr-1^+^CD11b^+^F4/80^+^ macrophages

**Fig. 3:**
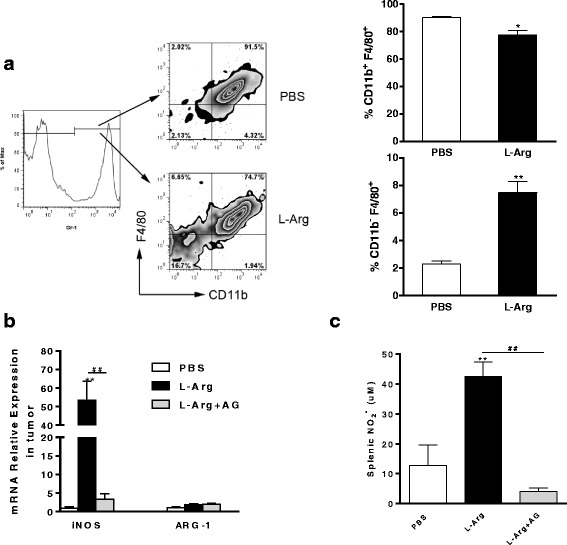
L-Arg affected two different populations of macrophages in the spleens of 4 T1 TB mice. Two populations of macrophages (CD11b^+^F4/80^+^ and CD11b^−^F4/80^+^ gated in Gr-1) were shown (**a**). Total RNA was purified from tumor block and ARG-1 and iNOS mRNA were quantified by real-time RT-PCR (**b**). NO levels from the supernatant of cultured splenic cells were evaluated by the Griess reaction (**c**). * *P <* 0.05 and ** *P <* 0.01 compared to PBS group. ^##^ *P <* 0.01 compared to L-Arg + AG group

To analyze whether L-Arg treatment influenced the frequency and functional state of macrophages, we first compared the percentages of two different populations of macrophages (CD11b^+^F4/80^+^ and CD11b^−^ F4/80^+^ gated in Gr-1) in the spleen. As shown in Fig. 3a, L-Arg treated TB mice produced a lower percentage of Gr-1^+^CD11b^+^F4/80^+^ macrophages (*P <* 0.05, *t-test*), but a higher percentage of Gr-1^+^CD11b^−^F4/80^+^ macrophages (*P <* 0.05, *t-test*) compared to control mice. Real-time RT-PCR analysis of the transcript levels of *iNOS* and *ARG-1* showed that L-Arg treatment significantly elevated the expression of *iNOS* (*P <* 0.05, *t-test*), compared with PBS and L-Arg + AG groups in tumor (Fig. 3b). *ARG-1* mRNA level was also elevated by L-Arg or L-Arg + AG treatment, but no statistically significance was detected between PBS and experiment groups (Fig. 3b). L-Arg treatment stimulated the macrophages to produce a significantly higher level of NO (*P <* 0.05, *t-test*) in the supernatant of cultured splenic cells than that in PBS and L-Arg + AG groups (Fig. 3c). No statistically significance between PBS and L-Arg + AG groups was detected in this experiment (Fig. 3b and c). These results demonstrated that L-Arg could decrease the numbers of immature macrophages, leading to elevated levels of iNOS mRNA and NO in 4 T1 TB mice.

### L-Arg promotes the differentiation and activation the of DCs in 4 T1 TB mice

**Fig. 4:**
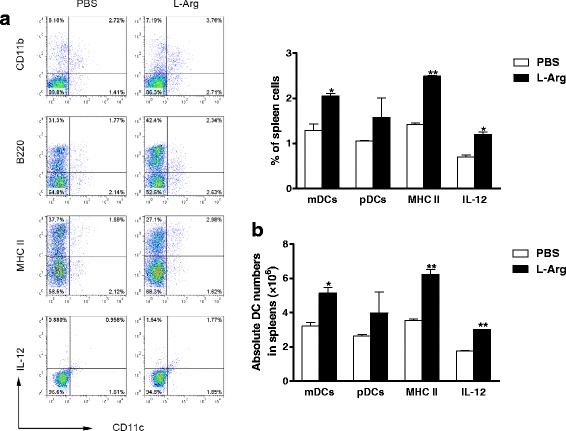
Effect of L-Arg treatment on DCs in 4 T1 TB mice. The frequencies (**a**) and absolute numbers (**b**) of mDCs, pDCs, CD11c^+^ MHC II^+^DCs, and CD11c^+^ DCs secreting IL-12 were determined in 4 T1 TB mice. Results are representatives of three independent experiments. * *P <* 0.05 and ** *P <* 0.01 compared to PBS group

Our next step was to determine whether the observed reduction in tumor mass was associated with enhanced immune responses elicited by L-Arg through the suppression of MDSCs. We first examined the L-Arg mediated regulation of the subsets and maturation of DCs late in breast cancer development. As shown in Fig. 4, there were higher percentages and numbers of myeloid DCs (mDCs, CD11c^+^ CD11b^+^) (*P <* 0.05, *t-test*) and plasmatocytoid DCs (pDCs, CD11c^+^ B220^+^) (*P <* 0.05, *t-test*) in the L-Arg supplement group than the control group. In addition, elevated maturation of CD11c^+^ DCs was observed, evidenced by the increased expression of MHC II (CD11c^+^ MHC II^+^) following L-Arg treatment (*P <* 0.05, *t-test*). Finally, increased frequency and number of CD11c^+^ DCs secreting IL-12 (CD11c^+^ IL-12^+^) at the end of L-Arg treatment was observed (*P <* 0.05, *t-test*). These results indicated that L-Arg treatment could initiate the expansion and activation of DCs.

### L-Arg promotes Th1 immune responses leading to inhibition of cancer development

**Fig. 5:**
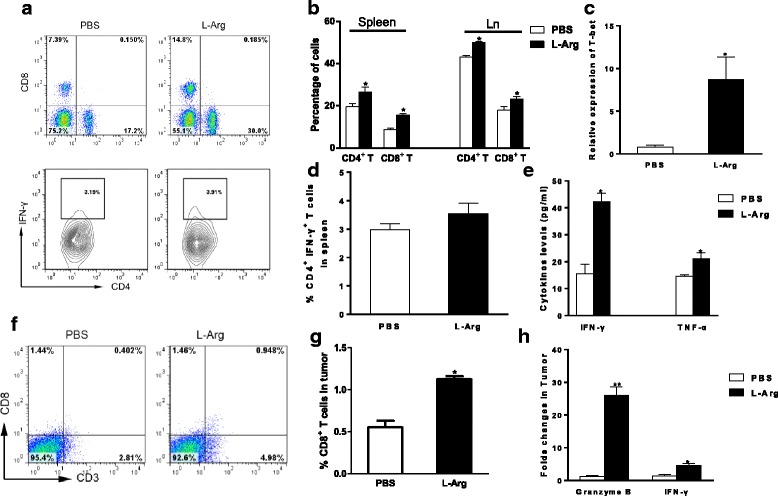
L-Arg promoted adaptive immune responses and inhibited cancer development. Flow cytometry analysis of CD4^+^ T cells, CD8^+^ T cells, and CD4^+^ IFN-γ^+^ T cells was shown (**a**, **b**, **d**, **f** and **g**). Total RNA was purified from spleen cells and T-bet mRNA were quantified by real-time RT-PCR (**c**). ELISA was used to determine the levels of TNF-α and IFN-γ (**e**). Total RNA was purified from tumor tissue and Granzyme B and INF-γ mRNA were quantified by real-time RT-PCR (**h**). * *P <* 0.05 compared to PBS group

In order to determine whether L-Arg treatment promoted Th1 immune response, the percentages of CD4^+^ and CD8^+^ T cells from lymph nodes and spleen were measured (Fig. 5a and b). The results showed that L-Arg increased the percentage of CD8^+^ cytotoxic T lymphocytes (CTLs) (*P <* 0.05, *t-test*) (Fig. 5f and g). We also found that L-Arg had no effect on the number of IFN-γ producing CD4^+^ T cells (CD4^+^ IFN-γ^+^) in the spleens of TB mice (Fig. 5d). However, the level of Th1 transcriptional factor T-bet significantly increased upon L-Arg treatment (Fig. 5c). Furthermore, as shown in Fig. 5e, TNF-α and IFN-γ levels in splenocyte supernatants were significantly increased in L-Arg treated TB mice compared to control mice (*P <* 0.05, *t-test*). Finally, we determined that the frequency of CTLs, mRNA levels of Granzyme B and IFN-γ in the tumor (Fig. 5h), and results showed L-Arg treatment significantly enhanced frequency and CTLs, and mRNA level of Granzyme B (a 10-fold increase compared with PBS) and IFN-γ (4-fold compared with PBS) significantly elevated. These results indicated that L-Arg supplementation promoted the development of adaptive immune response in TB mice.

### L-Arg has no effect on the Tregs in 4 T1 TB mice

**Fig. 6:**
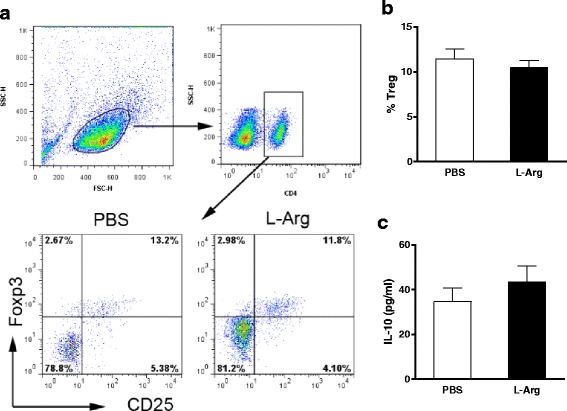
L-Arg had no effect on Tregs in 4 T1 TB mice. (**a**) and (**b**) We assessed the percentages of CD25^+^ Foxp3^+^ T cells gated on CD4 between the PBS and L-Arg group. (**c**) The level of IL-10 present in cultured splenic cell supernatants was assessed by ELISA. Results are representative of three independent experiments

As an important group of immune-suppressive cells, Tregs are considered to play a key role in the escape of tumor cells from host protective immune responses [19]. We next assessed whether L-Arg treatment could affect the number of Tregs (CD4^+^ CD25^+^ Foxp3^+^) in TB mice. The results from FACS analysis showed that L-Arg treatment had no effect on Tregs in TB mice (Fig. 6a and b). In addition, IL-10 levels produced by splenic cells were similar between the L-Arg and PBS groups, a result that was consistent with unaltered Treg levels (Fig. 6c). These data revealed that supplementation with L-Arg treatment had no obvious effect on Tregs in 4 T1 TB mice.

## Discussion

Mouse models are important tools to investigate the immune response and immunotherapeutic outcomes in cancer. In some experimental tumor models, L-Arg increases the latency period and survival rate, reduces tumor size and incidence, shortens the time of tumor regression, and inhibits tumor growth compared with other dietary interference or no dietary supplementation [20–22]. Dietary supplementation with L-Arg in patients with breast cancer significantly enhances host defenses [23, 24], and therefore may have a beneficial therapeutic role. In a related study, supplement of L-Arg significantly reduced the incidence of colorectal cancer due to a nonspecific stimulation of the host immune system [25]. In the present study, we supplemented 4 T1 TB mice with L-Arg and monitored anti-tumor immune responses. The results revealed that L-Arg prolonged survival time by inhibiting tumor growth. This was associated with the suppression of MDSCs and enhanced innate and adaptive immune responses. This suggests that L-Arg might be used as an adjuvant for breast cancer treatment.

MDSCs, typically positive for both CD11b and Gr1 in mice, are a population of immature myeloid cells defined by their suppressive actions on T cells, DCs, and natural killer cells. MDSCs can suppress T cell immune function via constitutive production of ARG-1, an enzyme responsible for significant L-Arg depletion [10, 26]. In addition to inhibiting T cells activation, MDSCs also impact anti-tumor immunity by perturbing innate immunity through their interactions with macrophages, NK cells, and NK T cells [27, 28]. Both MDSCs and T cells require L-Arg for protein synthesis. MDSCs produce high levels of intracellular arginase requiring them to import excess arginine through their CAT-2B transporter [29, 30]. As a result, they deplete L-Arg and limit L-Arg availability to T cells in the tumor microenvironment. Without L-Arg, naïve T cells in TB individuals cannot efficiently traffic to lymph nodes or tumor sites. MDSCs were found to infiltrate into tumors and promote tumor angiogenesis by producing high levels of MMP9 and by directly incorporating into tumor endothelium [31]. Hence, as a therapeutic target, down-regulation of MDSCs frequencies and/or abrogation of their immunosuppressive functions delay the tumor growth and prolong the survival both in animal models and in cancer patients [32–34]. Regulation of MDSCs includes the prevention of generation from bone marrow precursor cells and the stimulation of MDSCs differentiation towards mature DCs and macrophages. Therapeutic interventions targeting MDSCs may not only enhance the host immune system but also inhibit tumor invasion and metastasis [35]. In the present study, the frequencies of MDSCs were significantly suppressed in the 4 T1 TB mice after supplementation with L-Arg, and consistently, anti-tumor immunity was enhanced. Our results showed that L-Arg supplementation enhanced the anti-tumor immunity by suppressing the number of MDSCs in 4 T1 TB mice. This is in agreement with the recent report that L-Arg depletion blunted antitumor T-cell responses by inducing MDSCs [7]. Although the mechanism remains unclear, such inverse correlation between L-Arg and MDSCs may be mediated by the kinase GCN2, a key mediator of the effects induced by amino acid starvation [7]. In addition, several possible factors regulating MDSCs including VEGF, S100A8/A9, GM-CSF, and G-CSF may be involved in the downregulation of MDSCs by L-Arg supplementation. Dietary L-Arg was reported to decrease plasma VEGF [36]. More experiments are required to identify the key molecules to bridge the MDSCs and L-Arg in the breast cancer model in the future.

Macrophages, which are pivotal regulators in homeostatic tissue and tumor microenvironments, play dual roles during the progression of cancer. In one role, they activate and present tumor antigens to T cells, which are then activated to kill tumor cells [37]. At the same time, they release high levels of NO and ROS to kill tumor cells [38, 39]. On the other hand, as the immune surveillance is not sufficient anymore to prevent the occurrence of cancer, tumor-associated macrophages (TAM) contributes to tumor progression [40, 41]. Many observations indicate that TAM (Gr-1^+^ CD11b^+^ F4/80^+^) promote tumor progression and metastasis [42, 43]. In our study, flow cytometric analysis of splenic F4/80^+^ macrophages revealed that more than 90 % of the Gr-1^+^ cells had a CD11b^+^F4/80^+^ macrophage phenotype, which also was considered as a subset of MDSCs [42]. L-Arg treatment significantly decreased this population but elevated the frequency of CD11b^−^F4/80^+^ macrophages. In our study, L-Arg significantly elevated the mRNA level of iNOS, but not ARG-1, which was consistent with the higher level of NO. An earlier study showed that L-Arg could block the formation and development of colorectal tumors, and this effect might be related to the increased serum NO concentration and decreased ornithine decarboxylase activity [44]. Our results also indicated that L-Arg supplementation could significantly elevate the NO level in 4 T1 TB mice which was consistent with the increased level of CD11b^−^F4/80^+^ macrophages. However, NO was reported to have both deleterious and protective effects in the breast cancer [38, 45, 46]. Therefore, we further evaluated the role of NO in breast cancer by supplementation of an NOS inhibitor, aminoguanidine (AG) into the 4 T1 TB mice with L-Arg supplementation (L-Arg + AG). The results showed that L-Arg and L-Arg + AG treatment had a comparable suppressive effect on tumor tissue weight (data not shown), which reflects the complexity of NO in breast cancer. Considering the divergent cell sources of NO including macrophages [47], T cells [48], MDSCs [49], tumor cells [50], we speculate that the distinct sources and different bioavailability levels of NO may account for the inconsistent roles of NO in the tumor models. In addition, NO may serve as one but not the only one (such as IFN-γ, CTL) protective effector in the tumor bearing mice supplemented with L-Arg. Thus the exact role of NO in breast cancer needs to be explored in the future.

DCs play a pivotal role in bridging innate and adaptive immune responses. MDSCs decrease DC maturation, as well as the ability to take up antigen, migrate, and induce IFN-γ production in T cells [51]. In this study, immature DCs in TB mice were increased compared to the control group. A corroborating study showed that dietary supplementation with L-Arg enhanced T cell mediated immune function in healthy animals and human beings [52, 53]. We also found that L-Arg could promote the differentiation and activation of DCs in the spleen, which was associated with the initiation of the anti-tumor immune responses in TB mice. Our data showed that L-Arg treatment significantly increased the frequencies of mDCs and pDCs. IL-12 increases the capabilities of professional APCs in the tumor stromal and activates CD8^+^ T cells to detect antigen cross-presentation [54, 55]. In the 4 T1 model, IL-12 stimulates MDSCs to develop into mature myeloid cells. MDSCs obtained from tumors and spleens of tumor bearing mice treated with IL-12 up-regulated the surface markers of macrophages (F4/80 and MHC II) and DCs (CD80 and CD86) suggesting differentiation into more mature, less immunosuppressive forms. The spleens obtained from tumor-bearing mice also had up-regulation of many dendritic cell and macrophage maturation markers such as CD80, CD86, F4/80 and MHCII [56]. At the same time, high levels of IL-12 synthesized by mature DCs enhance both innate and acquired immunity [57, 58]. In this experiment, we found expression of MHC II and secretion of IL-12 by DCs were both significantly increased by L-Arg treatment.

L-Arg deprivation induces T cell hyporesponsiveness, as defined by profound reduction of T cell proliferation and reduced CD3ζ chain expression [6, 9]. Tumor-infiltrating CTLs have antitumor activity as judged by their favorable effect on patients’ survival and could potentially be exploited in the treatment of breast cancer [59]. However, T cells show anergy as both antigen-specific CD4^+^ and CD8^+^ T cells are tolerant to tumors. The mechanisms of CD8^+^ T cell tolerance to tumors include MDSCs [27] and Tregs [60]. MDSCs are also detected in tumor infiltrates and inhibit effector phase lytic functions of CD8^+^ tumor infiltrating lymphocytes [61]. A recent study showed that treatment of TB mice with 5-fluorouracil led to a major depletion of MDSCs in vivo but increased IFN-γ production by tumor-specific CD8^+^ T cells infiltrating the tumor and promoted T cell dependent antitumor responses in vivo [62]. These results indicated that therapy targeting MDSCs could be an effective method of cancer treatment. Our results demonstrated that L-Arg supplementation could reverse the immunosuppresive effects of MDSCs in 4 T1 TB mice as CD8^+^ T cells were significantly elevated within tumors. Undoubtedly, granzyme B is involved in an important pathway for CTL/NK cells-induced apoptosis [63], and L-Arg significantly elevated the mRNA level of granzyme B in tumor. Though CD4^+^ T cells producing IFN-γ was not increased, supplementation of TB mice with L-Arg elevate Th1 cells transcription factor T-bet, and also improved IFN-γ production. As a pro-inflammatory cytokine, IFN-γ induced surface expression of PD-L1 in breast cancer cells to induce the apoptosis of cancer cells [64].

In breast cancers, the percentage of Tregs, as assessed by Foxp3 positivity, increases in parallel with the disease stage [65, 66], indicating that the presence of Tregs promotes tumor progression through immunosuppression. IL-10 has been shown to modulate apoptosis and suppress angiogenesis during tumor regression [67, 68]. Here, our results showed that the level of Tregs transcription factor Foxp3 was significantly reduced upon L-Arg treatment.

In summary, L-Arg is an essential amino acid for promoting T cell function. However, the depletion of L-Arg by MDSCs in breast cancer patients or TB mice greatly reduces the anti-tumor immune responses. L-Arg supplementation in breast cancer bearing mice significantly decreased MDSCs as well as the ROS expression levels. This decrease was associated with enhanced innate and adaptive immune responses targeting tumors of the 4 T1 TB mice.

## Conclusion

Our results suggest that L-Arg supplementation may represent an effective adjunct therapy of breast cancer therapy to overcome immunosuppression mediated both by MDSCs and tumor cells to achieve better therapeutic effects in cancer patients.

## Abbreviations

AG, aminoguanidine; ARG-1, arginase 1; CT, cycle threshold; CTLs, cytotoxic T lymphocytes; DCs, dendritic cells; ER, estrogen receptor; FBS, fetal bovine serum; iNOS, inducible nitric oxide synthase; L, length; L-Arg, L-arginine; MDSCs, myeloid-derived suppressor cells; TB, tumor bearing; NO, nitric oxide; NOS2, nitric oxide synthase 2; Tregs, regulatory T cells; W, width
